# Palpable breast lumps: An age-based approach to evaluation and diagnosis

**DOI:** 10.4102/safp.v64i1.5571

**Published:** 2022-09-23

**Authors:** Francois Malherbe, Daniel Nel, Hunadi Molabe, Lydia Cairncross, Liana Roodt

**Affiliations:** 1 Department of Surgery, Faculty of Health Sciences, University of Cape Town, Cape Town, South Africa

**Keywords:** breast lump, palpable, primary care, diagnostic guide, age-based approach

## Abstract

A palpable breast lump is a common presentation of breast disease to a general practitioner. Fortunately, investigation of most of these lumps will lead to a benign diagnosis. It is essential to have a clear and systematic approach when investigating a palpable breast lump to avoid over investigation with the resultant increase in healthcare cost and anxiety. This article will discuss an approach to evaluating and diagnosing a palpable breast lump in the primary care setting.

## Introduction

Breast lumps are common in general practice. More than 25% of women are affected by breast disease during their lifetime, the vast majority of whom will complain of a new breast lump.^1^ Fortunately, most of these will be benign, with breast cancer only diagnosed in 10% of new breast lumps.^2^ It is essential to have an approach to investigating breast lumps that is simple to follow, limiting over investigation and preventing the misdiagnosis of malignant breast lesions.

The risk of a breast lump representing a breast cancer increases with age, with a 1/204 (0.49%) chance of breast cancer for a woman in her thirties, compared with 1/28 (3.54%) chance for a woman in her sixties.^3^ The risk of a breast lump being malignant in adolescence is exceedingly rare.^4^

## Triple assessment

With very few exceptions, every patient who presents with a breast lump needs to undergo a triple assessment. The triple assessment consists of a history and physical examination, some form of breast imaging and a biopsy. It is the gold standard in breast cancer diagnosis, with a positive predictive value of 100% if all three modalities are positive for breast cancer.^5^

### History and physical examination

A full history, as with any other patient, is important. When dealing with a breast complaint, appropriate history taking helps to risk-stratify the patient by identifying known factors associated with breast disease. This includes a detailed family history of cancer. A relevant family history in a patient with a breast lump includes asking about breast, ovarian, prostate, colon, pancreatic and other malignancies associated with specific genetic mutations, for example, BRCA 1 and 2, TP53, PALB 3, etc. Online risk calculators such as the Tyrer–Cuzick model (International Breast Cancer Intervention Study [IBIS], London, United Kingdom) help to individualise patient’s genetic risk.^6^ Information with regard to the patient’s oestrogen window (age of menarche and menopause), exogenous hormone exposure (use of contraceptives, hormone replacement therapy or fertility treatment), childbearing and breastfeeding history and lifestyle risk factors such as smoking, obesity and alcohol consumption further assist in the risk stratification process. It is also important to note any previous breast pathology or concerns. The physician should try to gain as much information as possible about the current complaint by enquiring about the characteristics of the lump; location and duration of the lump; changes in size; variation with menstrual cycle; pain, swelling or erythema; and nipple discharge or inversion.

A systematic examination of the breast must be undertaken. A visual inspection needs to be completed with the patient in a sitting and supine position. Observation must be made of the overall breast size, shape and symmetry. The best way is to ask the patient to position the ipsilateral hand under the head while the breast is examined. The examiner must use their finger pads, usually with the hand in a slightly cupped position. There are different techniques to examine a breast, but the most common methods used are the radial ‘wagon wheel’ method, the vertical strip method and the concentric circle method.^7^ Being thorough and consistently using the same approach is more important than a specific technique to achieve the best results, and different methods can be used on opposite breasts to make the examination ergonomically easier. The overall consistency of the breast is documented soft, firm or nodular. The position of any masses or tender lesions is observed in relation to the location in a conventional quadrant or clock face configuration. Essential findings to document would be characteristics of any abnormalities, including size, shape, texture, mobility, tenderness and approximate depth. Lastly, the examiner needs to examine the nipple–areola complex (NAC). The tissue around the NAC must be palpated to assess any abnormalities, including nipple discharge. The presence of a nipple discharge is noticed while the tissue around the areola is firmly pressed down. An axillary and supraclavicular fossa examination are part of a breast exam, and any palpable abnormalities in these areas must be documented. Should the clinician have difficulty in finding the reported lump, he or she can ask the patient to indicate where the lump was felt and in what position they were at the time the lump was felt. Some subtle changes may only be apparent if a patient is in a specific position, such as turned on the side or sitting up. Finding a breast lump with axillary adenopathy would raise high suspicion of breast cancer and needs a thorough assessment.

### Imaging

Radiological assessment of the breast is essential and commonly consists of either an ultrasound, mammogram or both. Another radiological test that is less typically used is a magnetic resonance imaging (MRI) of the breast. A breast MRI is usually carried out for one of the following reasons: to follow up high-risk patients with dense breast tissue or to obtain more information in selected patients diagnosed with breast cancer. It is advisable that a breast MRI should only be requested by a specialist. In women under 35 years, breast ultrasound would be the imaging modality of choice. An ultrasound examination can focus on the palpable lump to obtain more information to aid the diagnosis. However, the radiologist will usually scan the whole breast and axilla on both sides as part of routine breast ultrasound. Features on ultrasound that are consistent with a fibroadenoma include the following: (1) a well-defined homogeneously isoechoic or mildly hypoechoic solid lump with a maximum dimension of less than 30 mm; (2) an ovoid shape, lying parallel to the surface of the skin, with a smooth or gently lobulated contour; (3) a thin, echogenic pseudocapsule; and (4) lack of calcification and acoustic shadowing.^8^ Ultrasound is not hampered by breast density and is accurate for distinguishing between benign and malignant breast lesions, which are its main advantages compared with mammograms.^9,10^ Fibroadenomas are the most common lumps in young women, and ultrasound has been shown to have a sensitivity of 81.6% and specificity of 94.7% for making the diagnosis.^11^ After the age of 35 years, a mammogram of the breast is the investigation of choice, and ultrasound is used as an adjunct. The main drawback of mammograms is that they become less sensitive in detecting lesions in dense breasts, but tomograms, which consist of multiple mammograms at different angles, are helpful in dense breasts to diagnose small-size breast cancers. Most modern radiology practices have machines that have tomogram capability. Certain breast cancers can be missed by mammograms, either because the lump is out of view or the nature of the breast cancer makes them hard to see on a mammogram. It is important not to offer reassurance based on a normal mammogram but always to do a biopsy to complete the triple assessment in a patient with a palpable lump. The breast imaging reporting and data system (BI-RADS) is useful when interpreting mammogram and ultrasound results (Table 1).^12^ It aims to standardise breast imaging terminology, report organisation and assessment and provide a structured reporting classification system for mammography, ultrasound and breast MRI. The BI-RADS reporting method enables radiologists to communicate results to the referring physician clearly and consistently, with a final assessment and specific management recommendations. It consists of six categories, with each category providing a management suggestion and risk of breast cancer. Essentially, categories 1 and 2 are benign with a risk of malignancy approaching 0%. Category 3 recommends a short-term imaging follow-up in six months with a risk of malignancy of 2% or less. Categories 4 and 5 require a tissue diagnosis with a biopsy. The risk of malignancy for category 4 is between 2% and 95%, and the risk of malignancy for category 5 is more than 95%. Category 6 is not used often but is assigned when the patient is known with a malignancy.

**TABLE 1:** The BI-RADS 5th edition: Assessment categories and recommendations.

Category	Description	Recommendation	Management by General Practitioner
0	Assessment is incomplete: further evaluation is needed	Additional evaluation with mammographic views, ultrasound or (less commonly) breast MRI	Discuss with radiologist and book additional imaging
1	Negative: completely negative examination	Malignancy is not expected	Discharge
2	Benign findings: the imaging shows a benign lesion that carries no malignant potential	Malignancy is not expected	Discharge
3	Most likely a benign finding: controversial category to be used when a finding is almost certainly benign wherein a short interval follow-up is desired; unlikely to require biopsy and carries a chance of malignancy up to 2%	Follow-up examinations at short intervals of < 1 year (typically 6 months) for 24–36 months are recommended; stability seen at the end of follow-up is considered benign, at which point the finding is reassigned to category 2	Book follow-up imaging and examination in 6 months, continue to follow up for at least 24 months in patients older than 30 years
4	Suspicious abnormality: finding not classic for malignancy, > 2% to < 95% chance of malignancy	Some form of intervention is necessary, preferably image-guided core biopsy to establish a histopathologic diagnosis	Refer for ultrasound-guided biopsy and specialist surgical opinion
5	Highly suggestive of malignancy: 95% – 100%, the findings are characteristic of malignancy	Percutaneous core biopsy for tissue sampling to assist with management or to plan one-stage definitive surgical intervention, which may include lymph node sampling	Refer for ultrasound-guided biopsy and specialist surgical opinion
6	Known biopsy-proven malignancy: proven cancer	Appropriately used in patients who are undergoing neoadjuvant therapy or in those who require further staging; clinical management of the malignancy is recommended	Refer to a specialist for a surgical opinion

*Source*: Eghtedari M, Chong A, Rakow-Penner R, Ojeda-Fournier H. Current status and future of BI-RADS in multimodality imaging, from the AJR special series on radiology reporting and data systems. Am J Roentgenol. 2021;216(4):860–873. https://doi.org/10.2214/AJR.20.24894

### Biopsy

Initially, all breast lumps were diagnosed with an excision biopsy and histological assessment. This was replaced with fine needle aspiration biopsy (FNAB), a much less invasive diagnostic tool, in the 1960s. In the mid-1990s, ultrasound-guided core needle biopsies became available as an alternative. The current gold standard in diagnosing breast lumps is a core needle biopsy. It provides histology for an accurate diagnosis and allows immunohistochemistry (IHC) to be performed. Receptors on the surface of breast tumours are detected using IHC, and the receptor status is used as a surrogate to classify breast cancers into different types. The different types of breast cancers are managed differently, with some requiring surgery as the initial management, and in others, chemotherapy is given before surgery. Therefore, all breast cancers diagnosed with an FNAB require confirmatory core needle biopsy with IHC before a multidisciplinary team can make a treatment recommendation.

## Age-based approach to a palpable breast lump

Not every patient needs a full triple assessment. The risk of breast cancer increases with age, and a full triple assessment of every patient regardless of age would lead to an increase in costs at no benefit to the patient. Therefore, age has been proposed to guide investigations in the work-up of breast lumps (Table 2).^10,13,14^

**TABLE 2:** Common causes and tests required in the investigation of a breast lump based on age.

Age	Common causes	Tests	Red flags
≤ 19	Fibroadenoma cysts Hamartoma Fat necrosis Abscess	Clinical examination	Irregular firm mass Skin erythema or tethering Spontaneous bloody nipple discharge New onset nipple retraction Rapidly enlarging mass
≥ 20 to ≤ 24	Fibroadenoma Inflammatory breast conditions cysts Hamartomas	Clinical examination Ultrasound	
≥ 25	Fibroadenomas Inflammatory breast conditions cysts Breast cancer	Clinical examination Ultrasound Include mammogram if older than 40 years Core needle biopsy	

### Adolescence (under 20 years of age)

There are no statistics reporting the incidence of breast cancer in adolescents in South Africa, but according to the American Cancer Society, between 2012 and 2016, they reported an incidence of 0.1 per 100 000 in the United States.^4^ It is truly a one in a million event, and therefore healthcare providers need to take care not to over-investigate breast lumps in adolescence. The most common lump in adolescence is a fibroadenoma, representing 95% of breast lumps.^15^ Fibroadenomas, more than 5 cm in size in adolescents, are called giant juvenile fibroadenomas and are the most common indication for surgical intervention. Other less common lumps include cysts, hamartoma, fat necrosis or an abscess. Malignancies such as lymphomas or sarcomas could present as breast lumps in extremely rare cases. However, these lesions will have similar concerning features as malignant breast lesions and often grow rapidly, which should alert the treating physician. While consulting an adolescent, it is essential to enquire and look for possible red flags, which would include a significant family history of breast cancer under the age of 40, an irregular firm mass, skin erythema or tethering, bloody nipple discharge or nipple retraction and a rapidly enlarging mass. Adolescent patients need reassurance only if a typical, mobile 2 cm – 3 cm lump representing a fibroadenoma is found on clinical examination. A ruler or calliper can be used to document the size of the lump for follow-up comparison. A 6-month follow-up is appropriate to document no lesion enlargement. Imaging in the form of a breast ultrasound would be indicated if any red flags are present, and this would then be followed by a core biopsy, depending on the ultrasound result.

*Indication for referral:*

- red flag symptoms with ultrasound suggestive of breast cancer
- giant juvenile fibroadenoma.

### Patients between 20 and 25 years of age

Fibroadenomas are still the most common cause of a breast lump in these women, followed by inflammatory breast conditions, cysts and hamartomas. Although still extremely rare, some patients are diagnosed with breast cancer at this age.^14^ After a history and examination, an ultrasound would be indicated in all these women. In lesions that are solid and have the typical appearance of a fibroadenoma on ultrasound, no biopsy is indicated and reassurance with follow-up is all that is needed.^10,14,16^ The exact follow-up period is unknown, but in lesions with no red flags on history and examination and an ultrasound typical of a fibroadenoma, one ultrasound in 6 months to confirm the stability of the lesion is all that is needed. There is no need to remove fibroadenomas of less than 5 cm surgically. Only enlarging lesions or suspicious features on ultrasound would warrant an ultrasound-guided core biopsy to exclude breast cancer.

*Indication for referral:*

- red flag symptoms with ultrasound suggestive of breast cancer
- fibroadenomas of more than 5 cm in size
- palpable fibroadenomas smaller than 5 cm and the patient requests removal
- diagnosis of breast cancer.

### Patients older than 25 years of age

With an increase in age, the risk of breast cancer increases.^17^ Therefore, it is recommended that all women over 25 undergo a full triple assessment. Fibroadenomas are still common in patients between 25 and 30 years of age, but more breast cancers are diagnosed with increasing age.^13^ The safest approach would be to remove all palpable breast lumps in patients over 30 years. All lesions that are not excised need to be followed up by regular 6-monthly clinical examination and ultrasound to document stability for a minimum of two years.^18,19^

*Indication for referral:*

- patients between 25 and 30 years with a palpable lump who request removal
- patients older than 30 with a palpable lump
- diagnosis of breast cancer.

**FIGURE 1:**
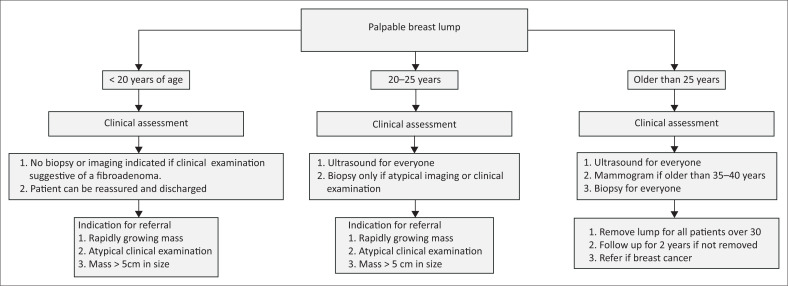
Algorithm for the management of palpable breast lumps based on the age of the patient.

Figure 1 represents a summary and diagnostic/treatment algorithm.

## Conclusion

Breast lumps are common and cause significant emotional stress for the patient when detected. An age-based approach to breast lumps allows for a cost-effective, safe and systematic pathway to investigate and treat breast lumps.
